# Disparities in care of older adults of color with cancer: A narrative review

**DOI:** 10.1002/cam4.6790

**Published:** 2024-01-17

**Authors:** Efrat Dotan, Shannon M. Lynch, Joanne C. Ryan, Edith P. Mitchell

**Affiliations:** ^1^ Department of Hematology/Oncology Fox Chase Cancer Center Philadelphia Pennsylvania USA; ^2^ Fox Chase Cancer Center Philadelphia Pennsylvania USA; ^3^ Pfizer Oncology Pfizer Inc. New York New York USA; ^4^ Clinical Professor of Medicine and Medical Oncology Sidney Kimmel Cancer Center at Jefferson Philadelphia Pennsylvania USA

**Keywords:** geriatric assessment, geriatric oncology, healthcare disparities, minorities, older adults, oncology

## Abstract

This review describes the barriers and challenges faced by older adults of color with cancer and highlights methods to improve their overall care. In the next decade, cancer incidence rates are expected to increase in the United States for people aged ≥65 years. A large proportion will be older adults of color who often have worse outcomes than older White patients. Many issues contribute to racial disparities in older adults, including biological factors and social determinants of health (SDOH) related to healthcare access, socioeconomic concerns, systemic racism, mistrust, and the neighborhood where a person lives. These disparities are exacerbated by age‐related challenges often experienced by older adults, such as decreased functional status, impaired cognition, high rates of comorbidities and polypharmacy, poor nutrition, and limited social support. Additionally, underrepresentation of both patients of color and older adults in cancer clinical research results in a lack of adequate data to guide the management of these patients. Use of geriatric assessments (GA) can aid providers in uncovering age‐related concerns and personalizing interventions for older patients. Research demonstrates the ability of GA‐directed care to result in fewer treatment‐related toxicities and improved quality of life, thus supporting the routine incorporation of validated GA into these patients' care. GA can be enhanced by including evaluation of SDOH, which can help healthcare providers understand and address the needs of older adults of color with cancer who face disparities related to their age and race.

## Graphical abstract

Older adults of color with cancer face healthcare disparities related to both their race/ethnicity and age. Many interconnected factors contribute to these disparities including clinical, age‐related physiologic, and treatment‐related factors, as well as the social determinants of health related to healthcare access, socioeconomic concerns, systemic racism, mistrust, and the neighborhood or built environment where a person lives. To allow for early identification of these factors and personalized management for older patients of color with cancer, we advocate for widespread implementation of an enhanced geriatric assessment that includes consideration of a patient's functional, cognitive, psychological, and nutritional status, their comorbidities, polypharmacy, and social determinants of health.

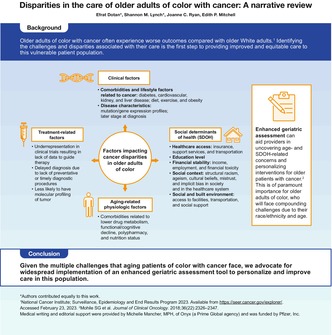

## 1 INTRODUCTION

It has been estimated that cancer incidence will increase by 67% from 2010 to 2030 for people aged 65 years or older in the United States (US). In the same time period, cancer incidence is also estimated to increase by 99% for racial and ethnic minorities of any age.^1^ With the aging population and the change in demographics of the total US population towards increased representation of ethnic minority groups, a greater number of older (≥65 years) adults of color are expected to face a cancer diagnosis.^1^ Given that adults of color have higher incidence rates and worse outcomes for many cancer types compared with White adults,^2^ new strategies are needed to ensure equitable care as this underserved patient population ages.

In this review, we discuss cancer disparities and factors contributing to inequities in the care of older adults of color with cancer. Identifying the needs and challenges of these patients is the first step to providing appropriate and personalized care and optimizing patient outcomes. Based on supporting evidence, we advocate for widespread implementation of an enhanced geriatric assessment (GA) to personalize and improve care and measures that support older adults and older adults of color with cancer.

## 2 CANCER DISPARITIES IN OLDER ADULTS OF COLOR: BACKGROUND

**FIGURE 1:**
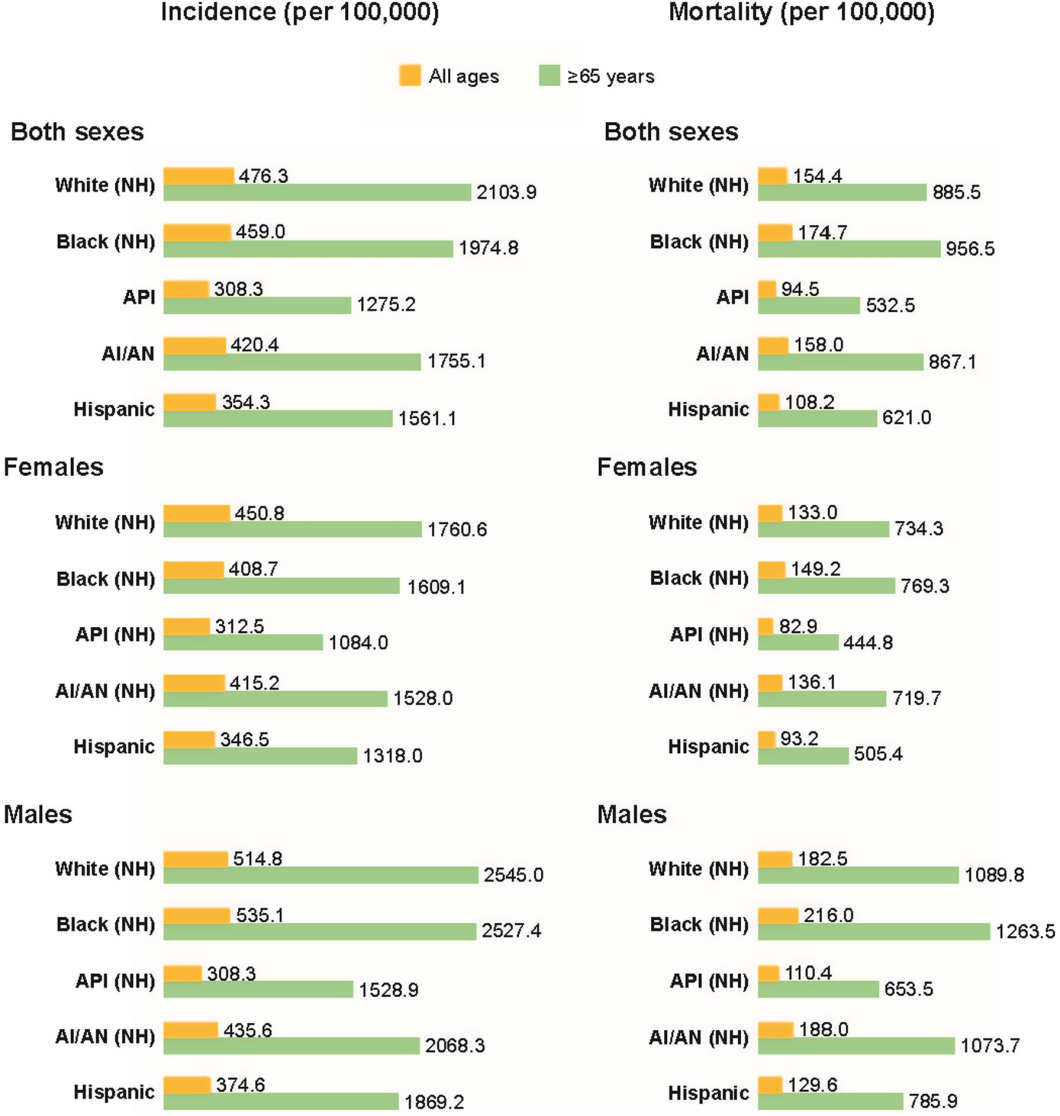
Overall cancer incidence (2015–2019) and mortality (2016–2020) rates (per 100,000) by race and ethnicity in the US for all ages and older adults (≥65 years). Abbreviations: AI/AN, American Indian/Alaskan Native; API, Asian/Pacific Islander; NH, Non‐Hispanic. *Source*: National Cancer Institute Surveillance Epidemiology, and End Results program SEER*Stat Database. Rates are per 100,000 and are age‐adjusted to the 2000 US standard population (19 age groups—Census P25‐1130). Rates for American Indians/Alaskan Natives only include cases that are in a purchased/referred care delivery area. Incidence data for Hispanics and non‐Hispanics are based on the North American Association of Central Cancer Registries Hispanic Latino Identification Algorithm.

Cancer disparities, described as differences in incidence, mortality/survival, screening rates, stage at time of diagnosis, and evidence‐based treatment,^3^, ^4^ between racial and ethnic groups in the US are well‐documented.^5^, ^6^ Overall cancer incidence and death rates are highest for Black men of all ages in the US (Figure 1). Analysis by cancer type demonstrates that incidence rates of liver and stomach cancers are between 1.6 and 2.2 times higher for Alaskan Native/American Indian, Asian or Pacific Islander, Hispanic, and Black patients compared with White patients. Additionally, Black patients experience more than double the incidence of myeloma compared with White patients (Table 1).^2^ While mortality rates from cancer have decreased across all racial and ethnic groups over the past decade, Black patients continue to experience the highest mortality rates both overall and for most cancer types^2^, ^7^; especially stomach, cervical, and multiple myeloma. Cancer‐related mortality rates of Alaskan Native/American Indian, Hispanic, and Asian or Pacific Islander adults are lower than those for White adults overall, but much higher for stomach and liver cancers.^2^

**TABLE 1:** Cancer burden disparities for racial and ethnic minority groups compared with the White population in the United States^a^.

	White (non‐Hispanic)	American Indian or Alaskan Native	Asian or Pacific Islander^b^	Hispanic (all races)	Black (non‐Hispanic)
Incidence rate ratio^c^					
All locations	1.00	0.73	0.65	0.73	1.00
Breast	1.00	0.69	0.77	0.70	1.00
Cervix uteri	1.00	1.23	1.02	1.47	1.48
Colon and rectum	1.00	0.96	0.82	0.82	1.22
Kidney and renal pelvis	1.00	1.20	0.53	0.99	1.13
Liver and intrahepatic bile duct	1.00	2.14	2.22	2.03	1.57
Lung and bronchus	1.00	0.72	0.58	0.47	1.05
Myeloma	1.00	0.97	0.65	1.06	2.29
Prostate	1.00	0.55	0.53	0.80	1.50
Stomach	1.00	1.64	1.99	1.88	1.93
Thyroid	1.00	0.70	0.95	0.84	0.57
Mortality rate ratio^d^					
All locations	1.00	0.90	0.64	0.78	1.22
Breast	1.00	0.82	0.53	0.71	1.39
Cervix uteri	1.00	1.45	1.05	1.20	2.26
Colon and rectum	1.00	1.03	0.72	0.81	1.37
Kidney and renal pelvis	1.00	1.46	0.50	1.02	1.22
Liver and intrahepatic bile duct	1.00	2.31	2.18	2.10	1.65
Lung and bronchus	1.00	0.77	0.56	0.47	1.10
Myeloma	1.00	1.07	0.64	1.10	2.35
Prostate	1.00	0.81	0.55	0.89	1.90
Stomach	1.00	1.86	1.90	1.91	2.01
Thyroid	1.00	1.10	1.02	1.13	1.03

^a^ *Source*: National Cancer Institute Surveillance Epidemiology, and End Results program SEER*Stat Database. Incidence and mortality data were analyzed using the Surveillance Research Program, National Cancer Institute SEER*Stat software. Data are shown as rate ratios between the White population and the population groups shown in the columns. Rates are per 100,000 and age‐adjusted to the 2000 US population. Rows indicate all cancer sites combined or individual cancer types.

^b^ Aggregated cancer mortality and incidence data are shown for the Asian or Pacific Islander population here.

^c^ Incidence rate ratio data shown are for 2018.

^d^ Mortality rate ratio data shown are for 2019.

Disparities in cancer screening rates between racial and ethnic groups have been reported by the Centers for Disease Control (Table 2)—even when screening rates are comparable, screening recommendations, patterns, and quality of screening techniques vary for different cancer types.^2^, ^8^, ^9^, ^10^ Black patients are generally diagnosed later in the disease course and have a higher likelihood of having advanced disease at diagnosis for cancers where screening is recommended (e.g., lung, colorectal, female breast, and cervical cancers), compared with other racial and ethnic groups.^11^ This may be due in part to the fact that screening guidelines do not take into account the increased prevalence of genetic mutations such as *PALB2* and *BRCA 1/2,* and age‐specific incidence for certain cancers in Black patients.^6^, ^12^

**TABLE 2:** Racial and ethnic disparities in screening rates for breast, colorectal, and cervical cancers in the United States, 2019.^9^

Race/ethnicity, %	Breast cancer	Cervical cancer	Colorectal cancer
White	76.0	77.9	69.8
Black	79.0	77.8	69.5
Hispanic	78.1	69.9	53.8
Asian	72.3	67.3	57.6

Studies in the US suggest that Black patients are less likely to receive evidence‐based care (e.g., guideline‐concordant care) than White patients for several types of cancers, including lung, colorectal, and breast cancers.^4^, ^6^ Additionally, a California‐based study found that Black, Hispanic, and certain Asian minority subgroups had an increased likelihood of delays in surgical treatment after a breast cancer diagnosis, compared with White patients.^13^

Cancer disparities between younger and older patients also exist. Older age is the greatest risk factor for cancer overall, as well as in many cancer types.^14^ Older patients with cancer have increased cancer‐related mortality and worse survival outcomes in comparison with younger patients across multiple cancer types, possibly related to higher rates of comorbidities and competing causes of death.^15^, ^16^, ^17^, ^18^ Additionally, although survival has improved in several cancers in recent years, these improvements have been less pronounced for older individuals.^19^ Older adults with cancer also often receive less intensive treatment (e.g., radiotherapy, chemotherapy, and surgery) compared with younger patients, regardless of their functional status or desire for more intensive care.^20^, ^21^, ^22^, ^23^

Several studies have assessed cancer disparities between racial and ethnic groups in older populations in the US; these studies have mostly focused on disparities between older Black and White patients.^16^, ^19^, ^24^, ^25^, ^26^, ^27^ Black patients (65–84 years) experience higher overall incidence of multiple simultaneous cancers, diagnoses at a later stage in the disease course, and lower survival rates compared with White patients of the same age range.^27^ Depending on the cancer type, disparities between racial groups can increase or decrease with age; for example, there is some evidence that differences in mortality between Black and White patients decrease with age in men with prostate cancer (i.e., improvements in survival were greater for older Black patients vs. older White patients) and increase for women with ovarian cancer (i.e., improvements in survival were smaller for older Black patients vs. older White patients).^19^

## 3 DETERMINANTS OF DISPARITIES IN OLDER ADULTS OF COLOR

**FIGURE 2:**
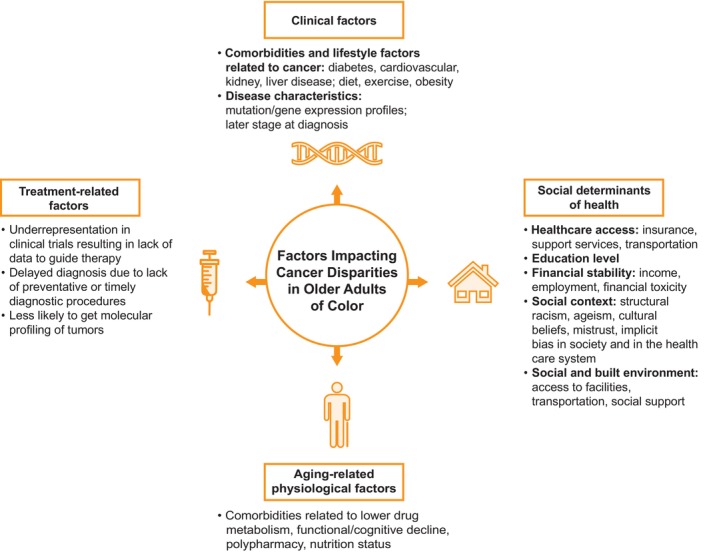
Multifactorial contributors to cancer disparities in older people of color.

The determinants of disparities in older adults of color are multifactorial and interconnected (Figure 2). Clinical and age‐related physiologic disparities can lead to treatment disparities. Social determinants of health (SDOH) and clinician bias can also impact these age and race/ethnic disparities associated with cancer diagnosis, treatment, and outcomes. Efforts by the National Comprehensive Cancer Network (NCCN), National Minority Quality Forum, and the American Cancer Society Cancer Action Network, as well as the American Society of Clinical Oncology (ASCO) have been ongoing to not only better understand and disentangle the main causes of these disparities, but identify strategies and recommendations for improving equity in cancer diagnoses, treatment, and outcomes.^28^, ^29^

### 3.1 Clinical factors

Older patients, patients of color, and patients who are part of both groups may have worse cancer outcomes due to clinical and biological factors, such as existing comorbidities, advanced stage of diagnosis, and different tumor biology. These clinical factors could lead to differences in available/recommended treatment options, response to therapy, and outcomes.

#### 3.1.1 Comorbidities

Comorbidities are highly prevalent among older adults with cancer; in an analysis of Medicare beneficiaries (aged >65 years), 67% with a cancer diagnosis had more than three other chronic conditions.^30^ Adults of color (in particular Black and some Hispanic patients) have an increased likelihood of having comorbidities, such as diabetes, cardiovascular disease, hypertension, and liver/kidney dysfunction compared with White patients.^2^, ^31^, ^32^, ^33^ These differences could be attributed to lifestyle and environmental factors, lack of access to quality care, and appropriate long‐term management of these conditions.

Patients with cancer and comorbidities have worse 5‐year survival outcomes compared with those without comorbidities across tumor types.^34^ This statistic may be related to polypharmacy, higher risk for drug–drug interactions, and nonadherence to cancer therapy.^17^, ^34^, ^35^, ^36^, ^37^, ^38^, ^39^ Furthermore, individuals with multiple comorbidities have a lower likelihood of receiving surgical intervention, radiation, and chemotherapy compared with patients without comorbidities, despite evidence showing a survival advantage associated with treatment, suggesting that undertreatment may also be an issue for these patients.^31^, ^35^

#### 3.1.2 Physiologic factors

In addition to comorbidities, physiologic changes with age in older adults (i.e., decreased kidney or liver function, and cognitive decline) can affect anticancer therapy and outcomes. Declines in kidney and liver function affecting drug metabolism, declines in kidney‐mediated drug elimination, changes in drug distribution due to higher body fat content, and reduction in plasma albumin leading to increased levels of unbound active drugs all potentially increase the chance of a person experiencing adverse reactions to drugs.^35^, ^39^ The impact of these factors can be mitigated by appropriate treatment adjustment based on thorough evaluation of kidney/liver function. Older adults are also at an increased risk of malnutrition (ranging from 3% in community settings to 29% in long‐term care settings^40^). Additionally, up to one third of people aged >65 years report hearing loss,^41^ and over one fifth of people ≥85 have age‐related visual acuity loss or blindness.^42^ Older age is also associated with cognitive decline; 7% of patients ≥68 years with cancer have diagnosed dementia and 36% of patients ≥70 years with advanced cancer report cognitive impairment.^43^, ^44^ These issues often are linked with poor treatment tolerance, poor adherence to treatment or supportive care measures, and additional challenges to providing cancer‐related care.^35^, ^45^, ^46^ These age‐related determinants of cancer susceptibility, prognosis, or response/tolerance to treatment can be exacerbated by socioeconomic factors (such as access to care or social support) that may vary in different racial/ethnic groups (see section 3.4).^2^

#### 3.1.3 Biologic features of tumors

Although there is limited research characterizing the molecular profile of most tumors for racial and ethnic minority groups, available evidence suggests that the type and frequency of inherited genomic variants vary across different racial/ethnic populations.^47^, ^48^, ^49^, ^50^ For example, a meta‐analysis looking at common mutations in people with lung cancer found that *EGFR* mutations occurred less frequently in Black patients compared with White, Asian, and Hispanic patients; additionally, *BRAF* mutations occurred less frequently in Black versus White patients and *ALK* mutations occurred less frequently in Black versus Hispanic patients.^51^ By contrast, *BRCA2* and *PALB2* mutations in breast cancer are thought to be more common in Black patients versus White (non‐Hispanic) patients.^12^ Furthermore, an analysis of the Cancer Genome Atlas found that breast cancers occurring in Black women had higher genomic instability and increased expression of cancer‐related signaling pathways compared with those occurring in White women.^52^ It is widely recognized that increased diversity in the reference human genome sequences used for molecular profiling is needed, so that germline genetic tests can become more applicable to patients of all races and ethnicities.^53^, ^54^ We should aim to improve our understanding of the genetic diversity among our patients to ensure that all patients are evaluated for possible targeted therapies, which would be highly desirable for older adults, given the better tolerance of these agents.

Although aging is a known cancer risk factor,^55^ the landscape of age‐associated changes in cancer mutational profiles remains largely uncharted for most tumors.^56^ A comprehensive genomic analysis of early‐ and late‐onset colon cancer found age‐related increases in the mutation rates of several genes (e.g., *ASXL1, BRAF*, and *CEBPA)* and specific common oncogenic mutations, such as *BRAF V600E*.^57^ A 2022 pancancer, genome‐wide study of age‐related differences across 35 tumor‐types found that older age impacts the number of somatic mutations as well as their evolutionary timing within a tumor, with specific age‐associated mutational signatures that reflect differences in underlying oncogenic processes.^55^ Despite existing evidence suggesting age‐related differences in tumor mutation burden, transcriptional profiles, and types of mutations,^55^, ^57^ large‐scale studies exploring the biologic basis for age‐based differences across tumor types remain limited.^55^ In particular, data are lacking on the association of cancer and aging as well as the impact of biologic differences for those cancers that occur in younger and older patients.^56^ Such studies would help us to better utilize existing treatment options as well as understand the options for personalization of therapy using targeted treatments for older adults, which may be better tolerated and provide improved outcomes over standard chemotherapy agents.

Despite evidence of differences in molecular profiles by race/ethnicity and age, the increasing importance of precision medicine, and recommendations by multiple medical society guidelines^58^, ^59^ for molecular profiling across multiple malignancies, many adults of color and older adults do not have these analyses performed on their tumors, thereby limiting treatment options and possibly affecting outcomes. Analysis of Medicare results from the Surveillance, Epidemiology, and End Results program found that among 5556 people with a lung cancer diagnosis, 26.2% of White patients and 32.8% of patients of Asian/other descent underwent genetic testing within 2 months of diagnosis compared with 14.1% of Black patients.^60^ Similarly disparate results are reported for molecular profiling for patients with ovarian cancer.^61^ A recent analysis of comprehensive genomic profiling in older adults with gastrointestinal cancers found that 55.7% of patients who underwent molecular profiling were aged <65 years, and patients aged ≥75 years were underrepresented, despite the existence of an age‐associated increase in high tumor mutational burden.^62^ Ensuring that older patients of color receive the latest standard of care, including molecular profiling, may help to mitigate disparities in cancer treatment and survival outcomes.

### 3.2 Lack of clinical trial representation

Clinical trials are crucial for the development of novel treatments but require adequate patient representation to provide physicians with appropriate guidance for treatment planning and recommendations.^2^, ^63^ Unfortunately, people of color and older adults have limited representation in cancer clinical trials.^64^, ^65^ In a study evaluating 230 trials that led to approval by the US Food and Drug Administration (FDA) of oncology drugs from 2008 to 2018, White patients represented 76.3% of trial participants versus 18.3%, 3.1%, and 6.1% for Asian, Black, and Hispanic patients, respectively.^64^ Similarly, only 24% of participants were ≥70 years of age in trials registered with the FDA, despite representing 42% of the cancer population.^65^ Furthermore, only 145 (63%) trials reported race, 18 (7.8%) evaluated the four major race groups in the US, and just 58 (25.2%) documented race subgroup analyses.^64^ Clinical trial underrepresentation contributes to disparate cancer outcomes, as providers are unable to anticipate how new treatments will affect populations of patients not included in the original trials.^2^, ^63^ Some providers may hesitate to use new therapies, leading to undertreatment; while others may overtreat patients, extrapolating clinical trial data and making unvalidated treatment adjustments.^66^ Strict eligibility criteria for therapeutic clinical trials are of particular concern. For example, excluding patients with certain comorbidities, such as cardiac, liver, or kidney dysfunction, can disproportionately impact people of color and older adults.^32^, ^67^ Interventions to minimize restrictive eligibility criteria may lead to more age and racial diversity in clinical trials.^2^, ^68^ Studies focusing on frail and vulnerable patient populations with comorbidities are also warranted. Furthermore, flexible and age‐focused clinical trial designs could increase participation of older adults.^63^ Incorporating pragmatic design elements should improve access to trials; specific examples include decreasing traveling time or helping patients get to trials, decreasing time spent in cancer centers, as well as bringing more trials to the community, where patients of color and older patients are typically seen.^69^ Ensuring the inclusion of these populations will provide useful information to providers caring for these patients, as well as helping to address disparities related to age and race/ethnicity. Regulatory agencies, such as the FDA, have guidance to increase representation in clinical trials among older adults and people of color, which may lead the way for improvements in clinical trial diversity.^63^, ^68^, ^69^

### 3.3 Biases, mistrust, and physician–patient communication

A national history of discriminatory practices, systemic inequalities, and structural barriers, often due to policies at societal and healthcare levels have led to differences in SDOH which are also associated with disparities observed by race/ethnicity and by age, and contribute to the disparities seen among older patients of color.^72^, ^73^, ^74^ On an individual level, implicit racial bias occurs unconsciously from internalized societal attitudes and stereotypes, and explicit racial bias occurs based on deliberate thought and judgments.^75^ Studies have shown that implicit bias among non‐Black oncologists was associated with shorter and less‐supportive interactions with Black patients.^76^ Personal experiences with racism among people of color may develop into diminished trust in healthcare providers and/or the system in general.^77^ Poor patient–provider relationships may be shaped by cultural aspects and/or language barriers.^2^, ^3^ Screening, prevention, and treatment implementation are all strongly influenced by the patient–provider relationship and trust in the medical system.^2^, ^3^ For instance, high levels of medical mistrust by Black patients have been linked with lower screening rates for colorectal cancer^78^ and reduction in quality of life in men with prostate cancer.^79^ Physician communication style can impact trust as well, with one study showing that poor physician communication led to Black patients with lung cancer having lower trust in their healthcare provider than White patients.^80^ Mistrust that results from implicit or explicit biases may also contribute to adults of color receiving less aggressive, inappropriate cancer treatments, as well as treatment delays, when compared with White patients.^2^, ^81^

Age‐based discrimination or ageism also exists in the healthcare system at the individual and system level. Health payment structures, lack of geriatric‐focused training, and underrepresentation in clinical trials are examples of health‐system level challenges for older patients.^82^ At the individual level, ageism encompasses negative attitudes, communication styles, and decision making from healthcare professionals towards older patients. For example, physicians can act patronizing towards their older patients and involve them less in their healthcare decisions, resulting in inferior outcomes.^83^, ^84^ Assumptions by providers of decline in cognitive function and frailty in older people with cancer may result in withholding appropriate therapy. Studies have demonstrated undertreatment and lower likelihood of receiving standard treatment in older versus younger patients.^83^ Lower digital literacy among older adults can also be misinterpreted as cognitive decline and exacerbate these issues.^82^ Providers should refrain from making assumptions regarding an older adult's goals for cancer therapy, which may differ from those of a younger person.^35^ For example, some older adults may value quality of life over longevity, especially if their functional status, cognitive abilities, or independence are impacted.^35^ Clinicians should discuss and understand their patients' goals and values by encouraging them to ask questions and share their hopes and concerns prior to making treatment decisions.^35^, ^45^ This is supported by findings of Mohile et al. who showed that inclusion of GA during oncology medical appointments for older patients with advanced cancer improved communication between patients and their healthcare providers about age‐related matters.^85^

Clearly, strategies establishing trust and minimizing bias among providers and older patients of color are needed to address systemic/institutional biases and to enhance care and cancer outcomes for older adults of color facing a cancer diagnosis. In addition, training programs must increase awareness and counter unconscious bias among providers to improve relationships with older people of color. Partnerships with leaders in the community and patient advocacy groups may raise awareness of preventive measures and available treatment options among older patients and minoritized communities.

### 3.4 Social determinants of health

Beyond clinical and biological indicators of disease, SDOH can also help explain disparities in both cancer incidence and outcomes by age and race/ethnicity. SDOH are the “conditions in the environments in which people are born, live, work, play, worship, and age that affect a wide range of health, functioning and quality‐of‐life outcomes and risks”.^70^ There are five key domains for SDOH: economic stability (e.g., opportunities for employment and income level); education access and quality (e.g., graduation rates for high school); social and community context (e.g., social/caregiver support, biases including structural racism, and ageism); healthcare access and quality (e.g., health insurance type and status, health literacy/language barriers, and proximity to facilities); and neighborhood and built environment (e.g., transportation options and socioeconomic status of community).^70^ SDOH have been found to influence treatment choices, outcomes, and contribute to the lack of participation of older patients and patients of color in clinical trials.^2^, ^71^

#### 3.4.1 Social support

Studies show that social support has a direct effect on the well‐being of a person with cancer, and their overall survival.^86^ Older adults, regardless of race, often depend on family members or other caregivers for home care, transportation, and medication assistance, among other physical and emotional support needs. In recent studies, nearly half of older adults with cancer reported having an unmet social support need,^87^, ^88^ with the most common unmet needs being medical and informational.^87^ Across all domains, patients with less education and household income, who were non‐White, had comorbidities, were never married or were divorced had significantly greater unmet needs. Conversely, there is some evidence to suggest that Black and Hispanic people may have improved social support and caregiving preparedness compared with White people, due to structural and environmental forces (e.g., multigenerational housing and low trust in health systems leading to increased reliance on social networks) increasing their use of close family and friend networks.^89^ Regardless, traditional provider assessments do not detect the support needs of their patients; for example, Seedor et al. showed that 73% of functional status, 44% of nutritional, and 96% of cognitive issues potentially requiring support were undetected by unstructured physician assessments among older metastatic breast cancer patients.^90^

#### 3.4.2 Economic stability and education

Economic stability and education are other important SDOH related to a patient's socioeconomic status. High cancer treatment costs place older adults and patients of color, who have a higher likelihood of living on a fixed/lower income, at increased risk of financial hardship, potentially leading to nonadherence to recommended treatment regimens and, ultimately, poorer outcomes.^2^, ^46^, ^91^ Socioeconomic concerns and rising costs may deter some people from fully utilizing healthcare services and receiving the proposed care. For example, Black and Hispanic patients with cancer are twice as likely to use financial coping behaviors, such as skipping medications, than White patients due to financial concerns.^2^

#### 3.4.3 Healthcare access and built environment

Access to healthcare is another significant challenge that people of color and older adults often face related to their socioeconomic status.^2^, ^3^, ^92^ Insurance status is linked closely to employment and is impacted by age and education, with a strong effect on patients' access to care. A study by Adamson et al. showed that providing health insurance coverage through Medicaid expansion reduced differences between Black and White patients in the time to first treatment for people with advanced cancers.^93^ While Medicare provides healthcare coverage for most older adults, some patients are not eligible and some services are not covered. Furthermore, even among Medicare beneficiaries, White adults have improved healthcare‐related experiences (e.g., getting appointments and care quickly) compared with patients of color.^94^ The built environment and, specifically, transportation barriers further affect patients' access to care and may impact patients' ability to comply with required follow‐up. Lack of driving, limited reliable, consistent, and affordable public transportation, and rural residence are common barriers to continuous treatment and cancer care for patients. In these cases, the financial burden of transportation and/or supportive care falls on the patient or their family, who may also have limited ability to face these financial hardships.^2^ These barriers also directly affect patients' access to clinical trials and novel treatment opportunities. Thus, interventions to address logistical barriers and reduce financial burden are warranted, and will result in significant improvement in patients' outcomes.

## 4 ADDRESSING DETERMINANTS OF DISPARITIES IN OLDER ADULTS OF COLOR: AN ENHANCED GERIATRIC ASSESSMENT

To begin to address these underlying determinants of disparity and provide comprehensive care to older adults, a thorough assessment is needed to identify all issues affecting the patient's care, treatment options, and outcomes, regardless of race/ethnicity or circumstance. Anticancer treatments are not contraindicated in older patients. A full GA should be performed to determine the true biological age and fitness of an older adult and to personalize treatments based on functional, cognitive, or psychosocial status, as well as comorbidities and social support (Table 3).^45^, ^83^, ^95^ Guidelines published by the ASCO,^96^ the NCCN,^45^ and the International Society of Geriatric Oncology (SIOG)^95^ call for GA use for treatment decisions for older adults with cancer.

**TABLE 3:** GA domains and rationale.^2^, ^35^, ^45^, ^96^, ^115^, ^127^

Domains^a^	Rationale for GA
Functional status	Poor functional status has been associated with lower survival rates, postoperative complications, and hospitalizations
Comorbidities	Comorbidities (e.g., diabetes, cardiovascular disease, and liver/kidney disease) may compromise treatment outcomes and survival, and are associated with chemotherapy toxicity and hospitalizations
Medication and polypharmacy	Polypharmacy can contribute to a higher risk of adverse drug reactions, drug–drug interactions, morbidity, and nonadherence to treatments
Nutritional status	Poor nutritional status is associated with treatment complications, increased mortality risk, poor chemotherapy tolerance (specifically hematologic toxicity), and an increased length of hospital stays
Cognition	Impaired cognition may lead to a reduced comprehension of treatments, slower recognition of side effects, and nonadherence to anticancer therapy These factors can contribute to poorer survival, functional dependence, depression, delirium, delayed diagnosis of complications, prolonged hospital stays, and increased chemotherapy toxicity
Psychological status	Quality of life and function may be negatively impacted by anxiety, depression, and distress Depression is associated with increased mortality, hospitalization, poor treatment tolerance, low social support, impaired cognition, polypharmacy, treatment noncompliance, and functional decline
Social status	Social isolation among older adults is a predictor of mortality and a risk factor for poor treatment tolerance and chemotherapy toxicity
SDOH	Inequities in factors related to SDOH (housing, education, language, healthcare access, transportation, and economic stability) contribute to disparities in exposure to cancer risk factors, behaviors, and outcomes

^a^ Association of Community Cancer Centers' “Practical Application of Geriatric Assessment: A How‐To Guide for the Multidisciplinary Care Team”^123^ provides the available tools for several domains.

Abbreviations: GA, geriatric assessment; SDOH, social determinants of health.

Multiple studies have demonstrated that GA can uncover issues that would influence treatment planning that are potentially missed by a routine clinical assessment as well as improve communication between providers and older patients.^35^, ^44^, ^85^, ^90^, ^97^, ^98^, ^99^ The use of GA allows the provider to obtain a more comprehensive understanding of a patient's overall health and social well‐being and adjust care accordingly. This approach is highly beneficial for older adults of all races and ethnicities. A meta‐analysis of observational and interventional studies assessing GA in patients with cancer found that GA revealed physical function deficits, malnutrition, depression, and comorbidities, and each of these variables were independently associated with chemotoxicity and/or overall survival.^100^ Similarly, a systematic review showed that GA promoted changes in cancer treatment plans for more than one quarter of patients, usually in favor of therapy deintensification, and recommended supportive care in over 70% of patients.^97^ A recent study demonstrated that older Black patients with newly diagnosed gastrointestinal cancer have increased frailty rates and limitations in function compared with their White counterparts, despite adjustment for multiple baseline characteristics.^101^

The gold standard Comprehensive GA is typically conducted by a geriatrics team and has been shown to decrease functional decline in inpatient settings and improve mental health in outpatient settings.^45^, ^95^, ^102^ The need for dedicated personnel and time limitations during clinic visits pose challenges for the incorporation of such a comprehensive assessment in the oncology outpatient setting.^103^, ^104^ As such, several tools have been created to help physicians identify patients at risk who would benefit from more thorough evaluation including Geriatric 8 (G8) and Vulnerable Elders Survey (VES‐13) (Table 4).^96^ An oncology‐specific GA was developed by the Cancer and Aging Research Group (CARG) and validated for use in clinical trials and routine practice.^105^ It includes self‐administered questionnaires for many domains, many of which are available in an electronic version facilitating incorporation into routine practice.^96^, ^106^ Each domain within the GA can be evaluated by multiple validated tools allowing the provider to choose the tool that they feel most comfortable with and compose a battery of assessments to fit their practice. The ASCO and the NCCN provide further guidance on the use of the various tools and incorporation of GA into routine practice.^45^, ^96^

**TABLE 4:** Geriatric and SDOH assessment tools.^35^, ^96^, ^105^, ^107^, ^108^, ^112^, ^113^, ^128^

Tool name	Description	Creator/organization and inks
G8 and VES‐13	Short, validated, screening tools that help to predict mortality in geriatric populations and can be used to identify patients in need of CGA	SIOG (G8): https://www.siog.org/files/public/g8_english_0.pdf ACCC (VES‐13): https://www.accc‐cancer.org/docs/projects/geriatric‐patients‐with‐cancer/how‐to‐guide/figure2_vulnerableelderssurvey_ves13.pdf?sfvrsn=db74c775_2
GA patient and healthcare professional tool and toxicity calculator	Oncology‐focused GA tools assessing functional status, comorbidities, medications, nutritional status, cognitive function, and psychosocial status. One tool is designed to be completed primarily by patients and one tool is designed for healthcare professionals. Toxicity calculator assessing sociodemographics, tumor/treatment variables, laboratory test results, and GA domains; including function, comorbidity, cognition, psychological state, social activity/support, and nutritional status	CARG: https://www.mycarg.org/?page_id=898
CRASH	Toxicity calculator that incorporates patient characteristics and chemotherapy regimen. The tool has components to be completed by a health provider (laboratory data, performance status, and chemotoxicity score) and portions to be completed by the patient (functional limitations, nutritional deficits, and mental health status)	Extermann et al. 2012 https://www.mdcalc.com/calc/10425/chemotherapy‐risk‐assessment‐scale‐high‐age‐patients‐crash‐score
PRAPARE®	SDOH questionnaire assessing personal characteristics, family and home, money and resources, and social and emotional health. It is designed to identify patients at risk of poor health outcomes	NACHC: https://prapare.org/the‐prapare‐screening‐tool/
AHC HRSN	SDOH questionnaire assessing housing instability, food insecurity, transportation problems, utility help needs, and interpersonal safety. It is designed to identify a patient's unmet needs that can be addressed through community intervention	CMS: https://innovation.cms.gov/files/worksheets/ahcm‐screeningtool.pdf

Abbreviations: ACCC, Association of Community Cancer Centers; AHC HRSN, Accountable Health Communities Health‐Related Social Needs; CARG, Cancer and Aging Research Group; CMS, Center for Medicare and Medicaid Services; CGA, comprehensive geriatric assessment; CRASH, Chemotherapy Risk Assessment for High‐Age Patients; G, geriatric; GA, geriatric assessment; NACHC, National Association of Community Health Centers; PRAPARE®, Protocol for Responding to and Assessing Patient Assets, Risks, and Experiences; SDOH, social determinants of health; SIOG, International Society of Geriatric Oncology; VES, Vulnerable Elders Survey.

Specific tools for the prediction of the risks for chemotherapy‐related toxicity for older adults have also been developed. The CARG chemotherapy toxicity calculator and Chemotherapy Risk Assessment Scale for High‐Age Patients (CRASH) are toxicity‐prediction calculators designed to evaluate the potential risk of grade 3–5 chemotherapy‐related toxicities for older patients with cancer.^35^, ^107^, ^108^ These calculators combine patient, disease, and treatment‐related factors to determine toxicity risk. This provides highly valuable information in personalizing the treatment plan and discussing the risk/benefit ratio with patients. However, these tools are not tumor specific; thus, research is ongoing to refine these tools and customize them to specific cancer types. For example, the CARG tool was recently adapted for breast cancer and refined to predict the risk of grade 3–5 chemotherapy‐related toxicity in older adults receiving chemotherapy for early‐stage breast cancer.^109^

A thorough GA, which covers multiple domains associated with the physical and social status of the patients, must also evaluate SDOH which strongly influence the care of older adults of color. A recent study found that 86% of social support issues identified among older adults with cancer using a GA were undetected by routine clinical assessment.^90^ As part of the social support evaluation of older patients, specific attention to the patient's housing situation, healthcare access, social support, education, and economic stability is warranted. Cancer treatments are expensive, and older patients, especially those on fixed income, can quickly reach their out‐of‐pocket maximum for spending resulting in financial toxicity. For patients without other support, this can lead to developing coping strategies such as stopping or skipping medications, and limiting doctor's visits or important diagnostic tests, which can negatively impact outcomes. Additionally, the stress associated with this financial toxicity, in and of itself, can lead to poorer quality of health.^110^ The answers to these social support questions can provide clinicians with key information to determine the need for additional and targeted supportive care services (i.e., patient navigation services) that can improve treatment adherence and outcomes.^111^

Validated tools for assessment of SDOH include the Protocol for Responding to and Assessing Patient Assets, Risks, and Experiences (PRAPARE®) and the Accountable Health Communities (AHC) Health‐Related Social Needs (HRSN) screening tool.^112^, ^113^ PRAPARE was designed to identify those at increased risk of having worse health outcomes utilizing a 17‐item questionnaire assessing at least one variable across four general domains (personal characteristics, family and home, money and resources, and social and emotional health). Additionally, PRAPARE includes four questions in the category of “Other”: specifically concerning incarceration, refugee status, abuse, and domestic violence.^112^ According to the 2019 Annual Medicaid Managed Care Survey, PRAPARE was deemed the most common social needs screening tool used by Medicaid Managed Care Organizations across the US, with a national engagement rate of 27%.^114^ However, a combined 50% of health centers continued to utilize internal or alternate systems, indicating a need for improved standardization of screening tools. The AHC HRSN screening tool developed by the Center for Medicare & Medicaid Innovation similarly utilizes a 10‐item questionnaire to understand patients' needs across five domains: housing instability, food insecurity, transportation problems, utility help needs, and interpersonal safety. An additional 16 questions across eight supplemental domains may be used to evaluate patients for family and community support, substance use, employment, financial concerns, mental health, education, physical functioning, and disabilities.^113^ Unlike other social needs screening tools, the AHS HRSN was specifically developed to identify HRSNs whose negative impact on patient health and healthcare utilization can be addressed through community interventions. It is intended for use by healthcare practitioners to support plans for treatment and referrals for community services, thereby potentially reducing overall healthcare utilization and improving patient outcomes.^115^

### 4.1 Overcoming barriers to GA in clinical practice

Despite the supporting evidence and guideline recommendations, GA is not widely used for patient evaluation, especially in community settings where most older adults of color are seen and treated.^104^, ^116^ Furthermore, studies have reported that most providers use unvalidated assessment tools for evaluating older adults and rely on the patient interview for determining an individual's fitness for therapy.^104^, ^117^ Providers cite multiple barriers to wider GA and SDOH assessment implementation (Table 5). Knowledge‐related barriers include unfamiliarity/confusion regarding the specific assessments or lack of training in this area.^104^, ^116^ While routine GA is not mandated for all patients, guidelines recommend a screening GA to identify patients who may need a more comprehensive assessment of their physical, cognitive, and emotional state.^45^, ^96^ These screening GAs, particularly those related to SDOH, may be administered in the context of telemedicine.

**TABLE 5:** Barriers to implementation of geriatric and SDOH assessments in oncology and potential solutions.^96^, ^103^, ^104^, ^111^, ^123^, ^129^

Barriers to GA and SDOH assessment implementation	Potential solutions
Lack of GA‐ or SDOH‐related knowledge or training (e.g., what GA and SDOH tools are available, when each tool should be used, and how to use them)	Training programs for healthcare providers on the use of GA and SDOH integration into clinical care Refer to guidelines and other websites (ASCO,^96^ SIOG,^129^ NCCN,^45^ ACCC,^123^ PRAPARE®,^112^ and AHC HRSN^113^)
Lack of resources (e.g., time, support staff, and referral options)	Use GA and SDOH tools that can be self‐administered or completed with a multidisciplinary team member and/or caregiver support Creation of multidisciplinary teams for geriatric care across multiple practices
Systemic barriers (e.g., reimbursement)	Increase awareness of GA and SDOH goals and benefits among policy makers Seek support from institutional stakeholders
Reluctance	Increase awareness of GA and SDOH goals and benefits among patients, caregivers, and the general population Partner with patient advocacy groups
SDOH barriers	Connect patients with resources (such as patient navigation programs) to address SDOH barriers (e.g., access to transportation and financial programs)

Abbreviations: ACCC, Association of Community Cancer Centers; AHC HRSN, Accountable Health Communities Health‐Related Social Needs; ASCO, American Society of Clinical Oncology; GA, geriatric assessment; NCCN, National Comprehensive Cancer Network; PRAPARE®, Protocol for Responding to and Assessing Patient Assets, Risks, and Experiences; SDOH, social determinants of health; SIOG, International Society of Geriatric Oncology.

Telemedicine may improve access to GA, particularly for older adults by enabling assessment, diagnosis, and treatment planning, while minimizing access barriers and exposure to pathogens.^118^, ^119^ In a systematic review of 17 randomized controlled trials, telemedicine procedures evaluated specifically for older adults have demonstrated adherence, patient acceptance, and improvement in patient health outcomes.^120^ This systematic review also observed substantial evidence supporting telemedicine in older adults as widely accepted, with three studies suggesting telemedicine may lead to improved cognitive function.^120^ However, the results were mixed for rural and low‐resource populations who may face challenges accessing these technologies, and better strategies are needed for implementing these programs in diverse settings. In addition, web‐based GA and SDOH methodologies, developed for integration with electronic medical records,^121^ have the potential to improve record keeping, portability, and access to clinically relevant GA data for clinicians.

Lack of resources, including time and personnel, is another barrier to GA implementation.^104^, ^116^ However, many tools can be self‐administered by patients or completed with caregiver support.^105^, ^122^, ^123^ CARG's primarily self‐administered oncology‐specific GA tool was successfully implemented in older adults with cancer in a cooperative group setting, with 87% of patients having completed their assessment without aid and with reporting overall satisfaction with the tool.^105^ Thus, one potential strategy for increasing the conduct of GA in rural and low‐resource settings may be to use local community centers or other settings with electronic capabilities as a shared resource to administer telehealth and web‐based/remote GA.

When GA abnormalities are identified, social workers, physical therapists, occupational therapists, or nutritionists can provide subsequent patient care and management.^45^, ^96^, ^124^ Depending on the health system, patient navigation services and social workers may be available to further address barriers to care driven by SDOH measures.^111^ Specialty services or patient navigation programs may be a significant barrier for small community practices that lack access to these services and see many older adults of color. Use of multidisciplinary teams shared across multiple practices and uniting resources for staff education and management may be helpful for community practices with limited support staff to deliver interventions.

Reimbursement issues, out‐of‐pocket costs, and patient or family misunderstanding or reluctance can also limit GA implementation.^103^, ^104^ Older patients who are not well‐informed about GA may have concerns regarding such evaluation, and worry about stigma and undertreatment of their condition.^103^ These obstacles are significantly greater and more difficult to overcome for older patients of color and compound existing multiple disparities and biases. Older people of color who are conscious of the inequities in cancer care may be even more inclined to doubt the value of GA. Language barriers and/or mistrust of healthcare systems may further hinder patient GA uptake. However, patient navigation programs can improve communication between patients and providers to help build interpersonal trust.^111^, ^125^ Wider education about the goals and benefits of GA is needed for key stakeholders, policymakers, community leaders, and especially patients and their caregivers. Efforts are underway to increase institutional awareness of the specific needs of the elderly and their caregivers, including the Age‐Friendly Health Systems program, which seeks to implement evidence‐based principles of high‐quality care for older adults in healthcare organizations.^126^ With strong evidence for GA‐guided care for older adults with cancer, providers must strive to overcome these barriers and incorporate GA into routine clinical practice.

## 5 CONCLUSION

Older adults and older adults of color face exceptional challenges and suffer from inequalities in cancer diagnosis and management, significantly affecting treatment outcomes. Efforts for reducing health disparities due to age and race, and providing equity of care, should be a nationwide top priority. As an initial step, better assessment of older adults of color using guideline‐recommended GA, enhanced with SDOH evaluations, will allow for early identification and management of health disparities for each patient and reduce the effect of disparities on patient care and outcomes. Improved knowledge of the barriers to and inconsistencies in the care of this vulnerable patient population, elimination of provider, and institutional bias, as well as implementation of support from multiple stakeholders, will allow us to achieve equity in care for older adults of color facing a cancer diagnosis and treatment.

## AUTHOR CONTRIBUTIONS

**Efrat Dotan:** Writing – original draft (equal); writing – review and editing (equal). **Shannon Lynch:** Writing – original draft (equal); writing – review and editing (equal). **Joanne C. Ryan:** Writing – original draft (equal); writing – review and editing (equal). **Edith Mitchell:** Writing – original draft (equal); writing – review and editing (equal).

## FUNDING INFORMATION

This work was supported by Pfizer Inc., New York, NY, USA.

## CONFLICT OF INTEREST STATEMENT

Shannon M. Lynch has received fees as a consultant with Beautycounter. Efrat Dotan has received grants or contracts from Incyte, Ipsen, Lilly, MedImmune, NGM Biopharmaceuticals, Relay Therapeutics, Lutris, Kinate, and Zymeworks; she has received consulting fees from Incyte, Helsinn, G1 therapeutics, Taiho, Olympus, and Amgen; and payment or honoraria from Pfizer. Joanne C. Ryan is an employee and shareholder of Pfizer, which provided funding for editorial support for the manuscript and is acknowledged above. Edith P. Mitchell has received grants or contracts from Bristol Myers Squibb, Exelixis, Genentech, GlaxoSmithKline, Pfizer, and Regeneron; she has received consulting fees from Astellas, Bristol Myers Squibb, Corvus, Genentech, and Janssen.

## ETHICS STATEMENT

This work is based on previously conducted studies and contains no new studies with human participants or animals performed by any of the authors.

## Data Availability

Data sharing does not apply to this article as no datasets were generated or analyzed during the current study.
